# Racial/Ethnic Differences in the Percentage of Gestational Diabetes Mellitus Cases Attributable to Overweight and Obesity, Florida, 2004-2007

**DOI:** 10.5888/pcd9.110249

**Published:** 2012-04-19

**Authors:** Shin Y. Kim, Lucinda England, William Sappenfield, Hoyt G. Wilson, Connie L. Bish, Hamisu M. Salihu, Andrea J. Sharma

**Affiliations:** Centers for Disease Control and Prevention; Division of Reproductive Health, National Center for Chronic Disease Prevention and Health Promotion, Centers for Disease Control and Prevention, Atlanta, Georgia; Bureau of Family and Community Health, Division of Family Health Services, Florida Department of Health, Tallahassee, Florida; Division of Reproductive Health, National Center for Chronic Disease Prevention and Health Promotion, Centers for Disease Control and Prevention, Atlanta, Georgia; Division of Reproductive Health, National Center for Chronic Disease Prevention and Health Promotion, Centers for Disease Control and Prevention, Atlanta, Georgia; Department of Biostatistics and Epidemiology, University of South Florida, Tampa, Florida; Division of Reproductive Health, National Center for Chronic Disease Prevention and Health Promotion, Centers for Disease Control and Prevention, Atlanta, Georgia

## Abstract

**Introduction:**

Gestational diabetes mellitus (GDM) affects 3% to 7% of pregnant women in the United States, and Asian, black, American Indian, and Hispanic women are at increased risk. Florida, the fourth most populous US state, has a high level of racial/ethnic diversity, providing the opportunity to examine variations in the contribution of maternal body mass index (BMI) status to GDM risk. The objective of this study was to estimate the race/ethnicity-specific percentage of GDM attributable to overweight and obesity in Florida.

**Methods:**

We analyzed linked birth certificate and maternal hospital discharge data for live, singleton deliveries in Florida from 2004 through 2007. We used logistic regression to assess the independent contributions of women's prepregnancy BMI status to their GDM risk, by race/ethnicity, while controlling for maternal age and parity. We then calculated the adjusted population-attributable fraction of GDM cases attributable to overweight and obesity.

**Results:**

The estimated GDM prevalence was 4.7% overall and ranged from 4.0% among non-Hispanic black women to 9.9% among Asian/Pacific Islander women. The probability of GDM increased with increasing BMI for all racial/ethnic groups. The fraction of GDM cases attributable to overweight and obesity was 41.1% overall, 15.1% among Asians/Pacific Islanders, 39.1% among Hispanics, 41.2% among non-Hispanic whites, 50.4% among non-Hispanic blacks, and 52.8% among American Indians.

**Conclusion:**

Although non-Hispanic black and American Indian women may benefit the most from prepregnancy reduction in obesity, interventions other than obesity prevention may be needed for women from other racial/ethnic groups.

## Introduction

Gestational diabetes mellitus (GDM), defined as carbohydrate intolerance leading to hyperglycemia first recognized during pregnancy, is associated with increased risk for pregnancy and delivery complications, including cesarean section, infant macrosomia, and neonatal hypoglycemia (1,2). Estimates of the prevalence of GDM among pregnant women in the United States range from 3% to 7%, depending on the population studied, the diagnostic tests employed, and data source used (3-5). Most women in whom GDM is diagnosed do not continue to have hyperglycemia after delivery (6). However, up to 50% of women with a history of GDM will develop type 2 diabetes in the decade following their GDM diagnosis (7).

In the United States, similar to racial/ethnic differences in type 2 diabetes, the risk of developing GDM is highest among Asian (particularly South Asian), black, American Indian, and Hispanic women (8-10), and these differences do not appear to be fully explained by differences in prepregnancy body mass index (BMI) (11,12). Obesity prevalence differs widely among women with GDM, being lowest among Asian women and highest among American Indian women. Although factors other than BMI, such as genetics and lifestyle, could be contributing to disparities in GDM risk (13,14), no studies have reported the population-attributable fraction (PAF) of GDM risk associated with elevated BMI by racial/ethnic group. A better understanding of the contribution of BMI status to GDM risk can help guide prevention efforts.

Florida is the fourth most populous US state and has high racial/ethnic diversity, making it a good source of data for studying racial/ethnic variations in the contribution of BMI status to GDM risk. The purpose of this study was to estimate the race/ethnicity-specific percentage of GDM attributable to overweight and obesity in the state of Florida from 2004 through 2007.

## Methods

We calculated the percentage of GDM attributable to overweight and obesity using Florida's revised live birth certificate data, which incorporate parts of the 2003 US Standard Certificate of Live Birth, which is linked to the Florida Hospital Inpatient Discharge database for live, singleton deliveries. We used data from March 2004 through December 2007 because these years were the only available that incorporated the 2003 US Standard and were linked to the Hospital Inpatient Discharge database. The University of South Florida in partnership with the Florida Department of Health linked the data sources. The investigators linked birth records to maternal inpatient hospitalizations using a multistage, stepwise approach; maternal Social Security number was the primary link. Women without a Social Security number (11.5% of births) were excluded, leaving 1,700,734 records to be potentially linked.

The investigators cleaned, standardized, and formatted all linking variables before linking data. They used date of delivery, facility of birth, infant's sex, and mother's residential zip code to improve or confirm the accuracy of the linkage. They were able to link 97.6% of unique singleton deliveries with known maternal Social Security numbers. The linked dataset represents all institutions in the state of Florida except for military and Veterans Affairs institutions. The human subjects coordinator deemed this study to be nonresearch, in accordance with the Centers for Disease Control and Prevention's (CDC's) Guidelines for Defining Public Health Research and Public Health Non-Research. Florida's Department of Health transferred de-identified data to CDC for analysis.

### Maternal characteristics

We used Florida's live birth certificate data to obtain maternal characteristics such as age, education, marital status, race/ethnicity, insurance status, parity, smoking status, birth country, prepregnancy BMI (based on weight and height data), and diabetes status during pregnancy. We based smoking status on the birth certificate item, "tobacco use during pregnancy," with 3 options: yes, no, and "yes, but quit." We categorized women who responded yes or "yes, but quit" as yes for any tobacco use.

Maternal race categories on Florida's birth certificate are white, black or African American, American Indian or Alaska Native, Asian Indian, Chinese, Filipino, Japanese, Korean, Vietnamese, other Asian, Native Hawaiian, Guamanian or Chamorro, Samoan, other Pacific Islander, and other. Maternal Hispanic and Haitian origin are collected separately on the birth certificate. Hispanic subcategories on the birth certificate include Mexican, Central/South American, Puerto Rican, Cuban, or other. For our main analysis, we grouped maternal race/ethnicity into 5 categories: non-Hispanic white, non-Hispanic black, Asian/Pacific Islander, American Indian, and Hispanic. Haitians were categorized according to their race. We also conducted ethnicity-specific analyses among women in the Asian/Pacific Islander group in which we grouped women as Asian Indian, Vietnamese, East Asian (Chinese, Korean, or Japanese), or Pacific Islander (Hawaiian, Filipino, Guamanian, Samoan, Pacific Islander). Women in the Hispanic group were grouped as Mexican, Puerto Rican, Cuban, or Central/South American. Data on Haitians were recombined and examined separately.

We calculated prepregnancy BMI (weight in kilograms divided by the square of height in meters) from the height and prepregnancy weight data obtained from the birth certificate. Florida recommends collecting data on prepregnancy weight and height for the birth certificate from the physician's prenatal record, labor and delivery records, or other hospital records at the time of delivery. For our main analysis, we used the National Heart, Lung, and Blood Institute's BMI categories (15) to classify women as underweight (<18.5), normal weight (18.5-24.9), overweight (25.0-29.9), class I obese (30.0-34.9), class II obese (35.0-39.9), or class III obese (≥40.0). Because people of Asian descent are at higher risk for diabetes and cardiovascular disease at lower BMI values than those of European descent (16), we used the following BMI categories recommended by the World Health Organization (17) for analysis of Asian/Pacific Islander groups: underweight (<18.5), normal weight (18.5-22.9), overweight I (23.0-24.9), overweight II (25.0-29.9), and obese (≥30.0).

To determine women's diabetes status during pregnancy, we used both birth certificate data and hospital discharge data. On birth certificates, women's diabetes status is indicated as prepregnancy diabetes (diagnosis prior to pregnancy), GDM (diagnosis during pregnancy), or no diabetes. Birth certificates on which both prepregnancy diabetes and GDM are indicated are sent back to the birth facility for corrections. In the Florida hospital discharge records, diabetes during pregnancy was identified by using the *International Classification of Diseases, Ninth Revision, Clinical Modification* (ICD9-CM) code 648.8 (abnormal glucose tolerance [GDM]) and codes 648.0 and 250.0 through 250.9 (diabetes mellitus [excludes GDM]). We used data from a medical record review of a small subset of the pregnancies in our linked dataset to formulate rules for assigning GDM status: GDM cases were deliveries in which hospital discharge data included the ICD9-CM code for gestational diabetes (648.8), except in instances in which the birth certificate record indicated preexisting diabetes.

Pregnancies without diabetes were those for which both the hospital discharge and birth certificate records indicated no diabetes (either preexisting or gestational). All other pregnancies (any deliveries where hospital discharge records indicated preexisting diabetes; where the birth certificate indicated some form of diabetes but the hospital discharge record indicated no diabetes; and hospital discharge record indicated both preexisting diabetes and GDM) were excluded from the analysis (2.0%). Using projections based on the medical records sample, we estimated that our classification methods had a sensitivity of 55.8% and a specificity of 99.9% in identifying women who had GDM and those who had no diabetes. We estimated that GDM misclassifications in this dataset would cause us to underestimate GDM prevalence by 44% but to underestimate relative risk (RR) and PAF by only about 5%, assuming nondifferential misclassification of GDM status.

We excluded the following women from the analysis: those with missing information on maternal prepregnancy BMI or race and Hispanic ethnicity; those that indicated more than 1 race/ethnicity; mothers with a height of less than 50 or greater than 77 inches, or a prepregnancy weight of less than 75 pounds; and those with diabetes status other than "GDM" or "no diabetes," according to our classification rules. After exclusions, our final dataset included 93.6% of deliveries from the linked dataset (n = 656,925).

### Statistical analysis

We described the distribution of maternal demographic and behavioral characteristics and estimated the prevalence and 95% confidence intervals (CIs) of GDM by BMI category overall and for each racial/ethnic group. Because of uncertainty about the make-up of the "other" racial/ethnic group, we excluded women in this group from all but the descriptive analyses. Our first criterion for including a variable as a potential confounder in our models was whether its inclusion changed the odds ratio by 10% or more. We included age because it satisfied this criterion for some (but not all) race groups. No other variable changed any odds ratio by as much as 10%. We also included parity as a potential confounder because it is associated with both BMI and GDM (18,19). To determine whether race/ethnicity modified the association between BMI and GDM, we tested interaction terms between BMI and race/ethnicity by using a likelihood ratio test (20).

Using the logistic regression results, we computed RRs and corresponding 95% CIs according to methods described by Flanders and Rhodes (21). We estimated the PAF and corresponding 95% CIs for each overweight and obese BMI category, for all overweight and obese categories combined overall, and by racial/ethnic groups. The overall PAF is equal to the sum of the categorical PAFs; these estimates were based on adjusted logistic regressions, according to methods described by Graubard and Fears (22). We also calculated the overall PAF standardized to the race distribution of the total US population. We interpreted each PAF estimate to be the reduction in GDM prevalence that would be expected to occur if all women in the overweight or obese BMI categories had a GDM risk equal to that of women in the normal BMI category, assuming that the risk for GDM among those with a low or normal BMI remained unchanged (23). Finally, we calculated the RR and PAF for Asian, Hispanic, and Haitian ethnic subgroups, using Asian-specific BMI categories for Asian subgroups.

We used logistic regression with locally weighted smoothing to estimate the probability of GDM as a continuous function of prepregnancy BMI. This analysis allowed us to evaluate the dose-response relationship between maternal BMI and GDM prevalence for each racial/ethnic group (24,25). Significance was set at *P* < .05.

## Results

The overall estimated prevalence of GDM was 4.7% and ranged by race/ethnicity from 4.0% among non-Hispanic blacks to 9.9% among Asians/Pacific Islanders (Table 1). GDM prevalence estimates were highest among Asians/Pacific Islanders across all BMI categories except for class III obesity, for which the estimate was highest among American Indians (Table 2). GDM prevalence was lowest among non-Hispanic blacks across all BMI categories.

**Figure 1.**
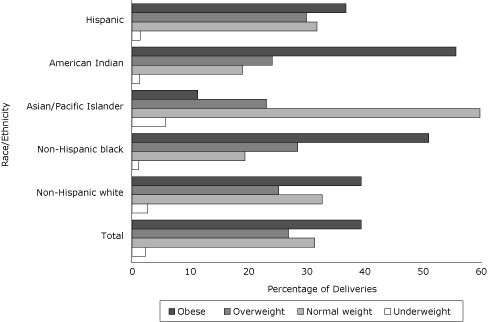
Percentage of deliveries among women with gestational diabetes mellitus in each body mass index (BMI) category, total and by race/ethnicity, Florida, 2004-2007. BMI categories (kg/m^2^) for all races/ethnicities defined by the National Heart, Lung, and Blood Institute (15) as follows: underweight, <18.5; normal weight, 18.5-24.9; overweight, 25.0-29.9; obese, ≥30.0.

Overall, 2.3% of women with GDM were underweight, 31.4% were normal weight, 26.9% were overweight, and 39.4% were obese (19.6% class I obese, 11.1% class II obese, and 8.7% class III obese) (Figure 1).

**Table:** 

Body Mass Index Category	Total	Non-Hispanic White	Non-Hispanic Black	Asian/Pacific Islander	American Indian	Hispanic

	%					
Underweight	2.3	2.7	1.1	5.7	1.3	1.4
Normal weight	31.4	32.7	19.4	59.8	19.0	31.8
Overweight	26.9	25.2	28.5	23.1	24.1	30.0
Class I obese	19.6	19.1	23.5	8.6	22.8	20.1
Class II obese	11.1	11.4	14.4	1.9	15.2	10.0
Class III obese	8.7	8.9	13.1	0.8	17.7	6.7

**Figure 2.**
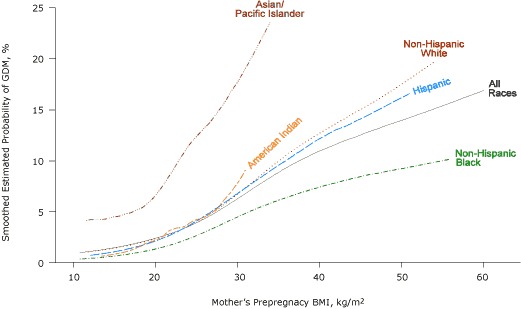
Smoothed estimated probability of gestational diabetes mellitus (GDM), by prepregnancy body mass index (BMI) and race/ethnicity, Florida, 2004-2007. BMI categories (kg/m^2^) defined by the National Heart, Lung, and Blood Institute (15) as follows: underweight, <18.5; normal weight, 18.5-24.9; overweight, 25.0-29.9; class I obese, 30.0-34.9; class II obese, 35.0-39.9; and class III obese, ≥40.0.

Compared with non-Hispanic white women with GDM, more non-Hispanic black women with GDM were overweight or obese and more Asian/Pacific Islanders women with GDM were underweight or normal weight (Figure 1). The probability of GDM increased with increasing BMI for all racial/ethnic groups (Figure 2).

There was no clear BMI threshold below which a dose-response relationship was not evident, although GDM risk increased only slightly with increasing BMI for BMI of less than 20.

The test for interaction between BMI and race/ethnicity was significant (*P* < .001). The adjusted RR for class III obesity was highest among American Indians (RR, 8.0; 95% CI, 4.1-15.8) and lowest among Asians/Pacific Islanders (RR, 3.0; 95% CI, 1.9-4.7) (*P* = .02) (Table 2). The adjusted percentages of GDM deliveries attributable to overweight and obesity were 41.1% overall, 15.1% among Asians/Pacific Islanders, 39.1% among Hispanics, 41.2% among non-Hispanic whites, 50.4% among non-Hispanic blacks, and 52.8% among American Indians (Table 2). The overall PAF standardized to the race distribution of the total US population in 2008 was 39.0%.

GDM prevalence estimates for Asian Indian (11.6%), Vietnamese (10.0%), East Asian (7.9%), and Pacific Islander women (9.8%) (Table 3) were all higher than those for any of the Hispanic subgroups (ranging from 4.4% among Cubans to 6.0% among Mexicans) (Table 4). The adjusted fractions of GDM deliveries attributable to overweight and obesity in Asian/Pacific Islander subgroups ranged from 9.1% in the Vietnamese subgroup to 34.0% in the Asian Indian subgroup (Table 3). PAF estimates for Hispanic and Haitian subgroups ranged from 30.3% among Central/South Americans to 48.1% among Haitians and Puerto Ricans (Table 4).

## Discussion

Our results suggest that if all women in Florida with a BMI of 25.0 or more had a GDM risk equal to that of women with a BMI in the normal range (18.5-24.9), 41.1% of GDM cases could be prevented. When we computed the corresponding PAF estimate standardized to the race/ethnicity distribution in the US population, the result was 39.0%. However, the contribution of BMI to GDM risk varied substantially by race/ethnicity. Although Asians/Pacific Islanders had the lowest percentage of GDM cases attributable to overweight/obesity, they had the highest GDM prevalence (9.9% overall and as high as 11.6% in Asian Indians). Even among underweight Asian/Pacific Islander women, GDM prevalence was 4.9%. In contrast, American Indians and non-Hispanic blacks had the 2 highest obesity rates, RRs, and PAF estimates, although non-Hispanic blacks had the lowest GDM rates. GDM prevalence and PAF estimates varied within Asian/Pacific Islander and Hispanic groups, depending on the subgroup ethnicity.

Our overall PAF estimate of 41.1% is consistent with results of an analysis of data from 7 states that participated in the Pregnancy Risk Assessment Monitoring System (PRAMS) from 2004 through 2007, which indicated that 46.2% of GDM cases were attributable to overweight and obesity (5). Furthermore, the dose-response relationship between BMI and GDM risk that we observed was consistent with what has been described for type 2 diabetes in the general population (26,27). However, to our knowledge, the prevalence of GDM across BMI categories in Asian/Pacific Islander, Hispanic, or Haitian subgroups has not been described in detail previously. Moreover, contrary to what has been reported in the literature (28), we found that non-Hispanic blacks had the lowest GDM prevalence of our 5 racial/ethnic groups.

Women with GDM are at increased risk for type 2 diabetes in later life. However, results from several randomized trials have demonstrated that increased physical activity and weight loss can reduce the risk for type 2 diabetes among women with a history of GDM (29); little is known about the effectiveness of such prevention interventions among Asian/Pacific Islander women with a history of GDM, particularly among those with a BMI in the normal range. Some evidence suggests that Asians in general may be more prone to insulin resistance than non-Hispanic blacks or non-Hispanic whites, which may be due to the difference in the distribution of fat stores between the groups and Asians' higher body fat percentages at given BMI levels (30). Therefore, traditional strategies for decreasing insulin resistance, such as high fiber consumption and increased physical activity, may be especially effective in this population (31).

To our knowledge, our study provides the first population-based race/ethnicity-specific estimates of the contribution of overweight and obesity to GDM prevalence. Using linked birth certificate and hospital discharge datasets is the best available approach to examine racial/ethnic disparities in the contribution of BMI to GDM risk at the population level.

Our study has limitations. Prepregnancy weight and height were obtained from birth certificates and may not be based on measurements obtained in clinical settings. Estimates of obesity prevalence based on self-reported height and weight tend to be lower than those based on measured height and weight. Although a previous study found minimal differences when comparing data on prepregnancy weight that were obtained from Florida birth certificates and from clinical measurements taken during the first trimester (32), we may have underestimated the prevalence of prepregnancy overweight and obesity, which would result in an underestimation of the RR and the PAF. Second, we likely underestimated GDM prevalence. However, assuming no bias occurred in sensitivity and specificity for GDM by BMI category, our analysis suggests that our RR and PAF estimates are biased slightly toward the null. Because the American College of Obstetricians and Gynecologists recommends universal GDM screening, we do not have any reason to believe that substantial bias occurred in GDM diagnosis in Florida, and ascertainment bias in GDM cases obtained by hospital staff for hospital discharge records by BMI seems unlikely. Third, we did not link approximately 11.5% of the birth certificate records with discharge records because of no maternal social security number. However, when we compared characteristics between the unlinked study population with the linked study population, we found that the unlinked women were more likely to be younger, be less educated, receive inadequate prenatal care, and be unmarried. Except for years of education, the differences were small, and the percentage of unlinked records was low, so the bias should be small. Fourth, we were not able to account for women who delivered more than once during the study period in our analysis (27%); however, we believe this would have produced only minor changes in our results. Fifth, we recognize that BMI is not a perfect measure of fat or central adiposity, but we were unable to examine any other indicators of adiposity. Racial/ethnic groups such as Hispanics and Asians are more prone to develop abdominal obesity, which has been considered a major contributor to the excessive prevalence of insulin resistance and diabetes (30). Finally, our data may not be generalizable to women in states other than Florida, to women whose pregnancies did not result in singleton live births, or to nonlegal residents.

Elevated prepregnancy BMI contributed substantially to GDM risk in most racial/ethnic groups, which suggests that decreasing the prevalence of overweight and obesity among women of reproductive age could substantially reduce the prevalence of GDM and associated delivery complications. However, the contribution of overweight and obesity to GDM risk varies by race/ethnicity and is considerably lower among Asians/Pacific Islanders and highest among American Indians and non-Hispanic blacks. Although non-Hispanic blacks and American Indians may benefit the most from a reduction in obesity, interventions other than obesity prevention may be needed for groups such as Asians/Pacific Islanders.

## Figures and Tables

**Table 1.** Demographic and Behavioral Characteristics of Women Who Had Live, Singleton Deliveries, Overall and by Race/Ethnicity, Florida, 2004-2007

**Characteristic**	Total (n = 656,925)	Non-Hispanic White, n (%) (n = 338,483)	Non-Hispanic Black, n (%) (n = 142,580)	Asian/Pacific Islander, n (%) (n = 16,799)	American Indian, n (%) (n = 1,252)	Hispanic, n (%) (n = 151,730)	Other, n (%) (n = 6,081)
**Age, y**							
<20	70,999 (10.8)	28,943 (8.6)	24,239 (17.0)	331 (2.0)	200 (16.0)	16,380 (10.8)	906 (14.9)
20-34	491,086 (74.8)	255,370 (75.4)	104,754 (73.5)	12,710 (75.7)	918 (73.3)	112,959 (74.5)	4,375 (71.9)
≥35	94,815 (14.4)	54,166 (16.0)	13,587 (9.5)	3,757 (22.4)	134 (10.7)	22,371 (14.7)	800 (13.2)
Missing	25	4	0	1	0	20	0
**Education, y**							
<12	111,092 (17.0)	44,049 (13.0)	33,728 (23.9)	1,351 (8.1)	446 (35.7)	30,529 (20.2)	989 (16.3)
12	210,501 (32.2)	97,656 (28.9)	57,500 (40.7)	3,287 (19.7)	403 (32.2)	49,810 (33.0)	1,845 (30.4)
>12	332,557 (50.8)	195,918 (58.0)	50,104 (35.5)	12,077 (72.3)	401 (32.1)	70,829 (46.9)	3,228 (53.2)
Missing	2,775	860	1,248	84	2	562	19
**Marital status**							
Married	378,184 (57.6)	228,502 (67.5)	42,516 (29.8)	14,307 (85.2)	560 (44.7)	89,159 (58.8)	3,140 (51.6)
Unmarried	278,723 (42.4)	109,971 (32.5)	100,060 (70.2)	2,492 (14.8)	692 (55.3)	62,567 (41.2)	2,941 (48.4)
Missing	18	10	4	0	0	4	0
**Insurance status**							
Medicaid	296,289 (45.2)	123,208 (36.5)	94,133 (66.3)	3,825 (22.9)	617 (49.4)	71,587 (47.3)	2,919 (48.2)
Private	321,896 (49.1)	198,968 (58.9)	40,640 (28.6)	11,330 (67.7)	521 (41.7)	67,667 (44.7)	2,770 (45.8)
None	28,578 (4.4)	11,004 (3.3)	5,721 (4.0)	1,273 (7.6)	87 (7.0)	10,293 (6.8)	200 (3.3)
Other	8,239 (1.3)	4,444 (1.3)	1,548 (1.1)	298 (1.8)	23 (1.8)	1,763 (1.2)	163 (2.7)
Missing	1,923	859	538	73	4	420	29
**Parity**							
0	279,875 (42.8)	150,981 (44.7)	53,526 (38.0)	7,625 (45.5)	413 (33.0)	64,411 (42.6)	2,919 (48.1)
1 or 2	313,660 (48.0)	162,493 (48.1)	65,204 (46.3)	8,275 (49.4)	574 (45.9)	74,509 (49.3)	2,605 (42.9)
>2	60,360 (9.2)	24,349 (7.2)	21,973 (15.6)	866 (5.2)	264 (21.1)	12,363 (8.2)	545 (9.0)
Missing	3,030	660	1,877	33	1	447	12
**Any smoking**							
Yes	67,179 (10.3)	54,375 (16.1)	6,867 (4.9)	357 (2.1)	204 (16.0)	4,593 (3.0)	786 (13.0)
No	588,513 (89.8)	283,222 (83.9)	135,504 (95.2)	16,431 (97.9)	1,051 (83.9)	147,015 (97.0)	5,290 (87.1)
Missing	1,233	886	209	11	0	122	5
**Country of birth**							
US	502,243 (76.5)	317,888 (94.0)	112,083 (78.7)	2,147 (12.8)	1,111 (88.8)	64,658 (42.6)	4,356 (71.7)
Other	154,408 (23.5)	20,465 (6.0)	30,403 (21.3)	14,643 (87.2)	140 (11.2)	87,037 (57.4)	1,720 (28.3)
Missing	274	130	94	9	1	35	5
**Body mass index,^a^ kg/m^2^**							
<18.5	34,918 (5.3)	20,164 (6.0)	6,091 (4.3)	1,915 (11.4)	52 (4.2)	6,320 (4.2)	376 (6.2)
18.5-24.9	336,122 (51.2)	184,430 (54.5)	58,277 (40.9)	11,119 (66.2)	507 (40.5)	78,572 (51.8)	3,217 (52.9)
25.0-29.9	154,725 (23.6)	73,495 (21.7)	37,908 (26.6)	2,794 (16.6)	349 (27.9)	38,785 (25.6)	1,394 (22.9)
30.0-34.9	75,991 (11.6)	35,109 (10.4)	21,610 (15.2)	741 (4.4)	197 (15.7)	17,728 (11.7)	606 (10.0)
35.0-39.9	33,393 (5.1)	15,560 (4.6)	10,587 (7.4)	165 (1.0)	95 (7.6)	6,672 (4.4)	314 (5.2)
≥40.0	21,776 (3.3)	9,725 (2.9)	8,107 (5.7)	65 (0.4)	52 (4.2)	3,653 (2.4)	174 (2.9)
Missing	0	0	0	0	0	0	0
**Gestational diabetes mellitus**							
Yes	30,455 (4.7)	15,456 (4.7)	5,522 (4.0)	1,608 (9.9)	79 (6.5)	7,502 (5.1)	288 (4.9)
No	611,242 (95.3)	315,670 (95.3)	133,322 (96.0)	14,620 (90.1)	1,132 (93.5)	140,873 (94.9)	5,625 (95.1)
Missing	15,228	7,357	3,736	571	41	3,355	168

a Body mass index (kg/m^2^) categories defined for all races/ethnicities by the National Heart, Lung, and Blood Institute (15) as follows: underweight, <18.5; normal weight, 18.5-24.9; overweight, 25.0-29.9; class I obese, 30.0-34.9; class II obese, 35.0-39.9; and class III obese, ≥40.0.

**Table 2:** Prevalence, Relative Risk, and Population-Attributable Fraction of Gestational Diabetes Mellitus, by Race/Ethnicity, Florida, 2004-2007

BMI,^a^ kg/m^2^	All Races^b^	Non-Hispanic White	Non-Hispanic Black	Asian/Pacific Islander	American Indian	Hispanic
**Prevalence, % (95% CI)**						
<18.5	2.0 (1.9-2.2)	2.1 (1.9-2.3)	1.0 (0.8-1.3)	4.9 (3.9-5.9)	2.0 (0-5.8)	1.7 (1.4-2.0)
18.5-24.9	2.9 (2.8-3.0)	2.8 (2.7-2.9)	1.9 (1.8-2.0)	8.9 (8.4-9.4)	3.0 (1.5-4.5)	3.1 (3.0-3.2)
25.0-29.9	5.4 (5.3-5.5)	5.4 (5.3-5.6)	4.2 (4.0-4.5)	14.1 (12.7-15.4)	5.6 (3.2-8.1)	5.9 (5.7-6.2)
30.0-34.9	8.2 (8.0-8.4)	8.7 (8.4-9.0)	6.3 (5.9-6.6)	20.2 (17.2-23.2)	9.6 (5.4-13.8)	8.8 (8.4-9.3)
35.0-39.9	10.7 (10.3-11.0)	11.9 (11.3-12.4)	7.9 (7.4-8.4)	20.4 (14.0-26.8)	13.6 (6.5-20.8)	11.8 (11.0-12.6)
≥40.0	13.0 (12.6-13.5)	15.1 (14.4-15.8)	9.6 (8.9-10.2)	24.1 (12.7-35.5)	28.0 (15.6-40.4)	14.8 (13.6-16.0)
**Adjusted^c^ relative risk (95% CI)**						
<18.5	0.7 (0.7-0.8)	0.9 (0.8-0.9)	0.6 (0.5-0.8)	0.6 (0.5-0.7)	0.7 (0.1-5.3)	0.6 (0.5-0.8)
18.5-24.9	1 [Reference]					
25.0-29.9	1.9 (1.9-2.0)	1.9 (1.9-2.0)	2.0 (1.9-2.2)	1.6 (1.4-1.8)	1.8 (0.9-3.4)	1.8 (1.7-2.0)
30.0-34.9	3.0 (2.9-3.1)	3.1 (3.0-3.3)	2.9 (2.7-3.2)	2.3 (2.0-2.7)	3.1 (1.6-6.0)	2.8 (2.6-2.9)
35.0-39.9	3.9 (3.8-4.1)	4.3 (4.0-4.5)	3.7 (3.4-4.0)	2.4 (1.8-3.3)	4.2 (2.0-8.8)	3.7 (3.5-4.0)
≥40.0	4.9 (4.7-5.1)	5.4 (5.1-5.7)	4.5 (4.1-4.9)	3.0 (1.9-4.7)	8.0 (4.1-15.8)	4.7 (4.3-5.1)
**Adjusted^c^ population-attributable fraction (95% CI)**						
25.0-29.9	12.8 (12.2-13.5)	12.2 (11.3-13.0)	14.4 (12.8-16.0)	8.5 (6.2-10.8)	10.4 (−1.9-22.7)	13.7 (12.4-15.1)
30.0-34.9	13.0 (12.5-13.5)	13.0 (12.4-13.7)	15.4 (14.1-16.7)	4.9 (3.6-6.3)	15.4 (5.0-25.7)	12.8 (11.8-13.8)
35.0-39.9	8.3 (7.9-8.7)	8.7 (8.2-9.2)	10.5 (9.5-11.4)	1.1 (0.5-1.8)	11.6 (3.4-19.8)	7.3 (6.6-8.0)
≥40.0	6.9 (6.6-7.3)	7.2 (6.8-7.7)	10.2 (9.3-11.1)	0.5 (0.1-1.0)	15.4 (7.1-23.8)	5.3 (4.7-5.8)
Total (≥25.0)	41.1 (40.2-42.0)	41.2 (39.9-42.4)	50.4 (47.9-53.0)	15.1 (12.3-17.9)	52.8 (32.4-73.2)	39.1 (37.2-41.1)

Abbreviation: BMI, body mass index; CI, confidence interval.

a BMI (kg/m^2^) categories defined for all races/ethnicities by the National Heart, Lung, and Blood Institute (15) as follows: underweight, <18.5; normal weight, 18.5-24.9; overweight, 25.0-29.9; class I obese, 30.0-34.9; class II obese, 35.0-39.9; and class III obese, ≥40.0.

b The "All Races" group does not include "other" race.

c Models are adjusted for age and parity. Models for the "All Races" group are adjusted for race.

**Table 3.** Prevalence, Relative Risk, and Population-Attributable Fraction of Gestational Diabetes Mellitus, by Asian Racial/Ethnic Subgroup, Florida, 2004-2007

**BMI,^a^ kg/m^2^**	Asian Indian	Vietnamese	East Asian^b^	Pacific Islander^c^
**Prevalence, % (95% CI)**				
<18.5	4.7 (2.5-7.0)	5.8 (3.9-7.8)	3.8 (2.1-5.4)	6.1 (3.4-8.8)
18.5-22.9	7.8 (6.6-9.0)	9.8 (8.4-11.2)	6.7 (5.6-7.9)	7.0 (5.9-8.2)
23.0-24.9	14.6 (12.3-17.0)	14.5 (10.4-18.5)	12.5 (9.2-15.7)	10.1 (7.9-12.4)
25.0-29.9	13.9 (11.8-16.1)	15.1 (10.3-19.8)	12.3 (8.9-15.7)	14.2 (11.7-16.7)
≥30.0	24.3 (19.8-28.9)	13.6 (3.5-23.8)	17.8 (9.9-25.7)	19.5 (15.0-24.0)
Overall	11.6 (10.6-12.5)	10.0 (8.9-11.1)	7.9 (7.0-8.8)	9.8 (8.9-10.7)
**Adjusted^d^ relative risk (95% CI)**				
<18.5	0.6 (0.4-1.0)	0.6 (0.4-0.9)	0.6 (0.4-1.0)	0.9 (0.6-1.5)
18.5-22.9	1 [Reference]			
23.0-24.9	1.9 (1.5-2.3)	1.4 (1.1-2.0)	1.8 (1.3-2.5)	1.4 (1.1-1.9)
25.0-29.9	1.8 (1.4-2.2)	1.5 (1.1-2.1)	1.8 (1.3-2.5)	2.0 (1.6-2.5)
≥30.0	3.1 (2.4-3.9)	1.4 (0.7-3.0)	2.7 (1.7-4.3)	3.0 (2.3-4.0)
**Adjusted^d^ population-attributable fraction (95% CI)**				
23.0-24.9	11.5 (7.0-16.0)	4.6 (0.2-8.9)	8.7 (3.5-14.0)	5.2 (0.8-9.6)
25.0-29.9	11.6 (6.8-16.4)	3.9 (0.1-7.8)	7.7 (2.7-12.6)	13.8 (8.6-19.0)
≥30.0	11.0 (7.7-14.2)	0.6 (−1.0-2.2)	3.9 (1.1-6.8)	10.1 (6.5-13.7)
Total (≥23.0)	34.0 (25.8-42.2)	9.1 (2.8-15.4)	20.3 (12.2-28.4)	29.1 (20.8-37.5)

Abbreviations: BMI, body mass index; CI, confidence interval.

a BMI (kg/m^2^) categories defined by the World Health Organization (17) for analysis of Asian/Pacific Islander groups as follows: underweight, <18.5; normal weight, 18.5-22.9; overweight I, 23.0-24.9; overweight II, 25.0-29.9; and obese, ≥30.

b East Asian: Chinese, Korean, Japanese.

c Pacific Islander: Hawaiian, Filipino, Guamanian, Samoan, Pacific Islander.

d Models are adjusted for age and parity.

**Table 4.** Prevalence, Relative Risk, Population Attributable Fraction of Gestational Diabetes Mellitus, by Hispanic and Haitian Racial/Ethnic Subgroup, Florida, 2004-2007

**BMI,^a^ kg/m^2^**	Mexican	Puerto Rican	Cuban	Central/South American	Haitian
**Prevalence, % (95% CI)**					
<18.5	2.1 (1.1-3.1)	1.7 (1.1-2.4)	1.6 (0.9-2.2)	1.6 (1.0-2.2)	1.5 (0.5-2.5)
18.5-24.9	3.1 (2.8-3.5)	2.7 (2.4-2.9)	2.8 (2.5-3.0)	3.2 (3.0-3.5)	2.6 (2.3-3.0)
25.0-29.9	6.6 (6.0-7.2)	6.2 (5.7-6.7)	5.3 (4.9-5.8)	5.6 (5.1-6.0)	6.4 (5.8-7.0)
30.0-34.9	9.1 (8.1-10.1)	9.3 (8.4-10.1)	7.7 (6.8-8.5)	8.8 (7.9-9.7)	9.3 (8.2-10.3)
35.0-39.9	12.7 (10.9-14.5)	12.9 (11.4-14.4)	9.5 (7.9-11.0)	10.9 (9.1-12.7)	11.6 (9.6-13.6)
≥40.0	16.5 (14.0-18.9)	14.9 (12.8-17.0)	14.8 (12.1-17.5)	11.1 (8.4-13.9)	8.2 (5.4-11.1)
Overall	6.0 (5.7-6.4)	5.3 (5.0-5.5)	4.4 (4.2-4.7)	4.6 (4.4-4.8)	5.4 (5.1-5.7)
**Adjusted^b^ relative risk (95% CI)**					
<18.5	0.8 (0.5-1.3)	0.8 (0.5-1.1)	0.7 (0.4-1.0)	0.5 (0.4-0.8)	0.6 (0.3-1.2)
18.5-24.9	1 [Reference]				
25.0-29.9	1.9 (1.7-2.2)	2.1 (1.9-2.4)	1.8 (1.6-2.1)	1.7 (1.5-1.9)	2.1 (1.8-2.5)
30.0-34.9	2.6 (2.2- 3.0)	3.2 (2.8-3.6)	2.6 (2.3-3.0)	2.7 (2.4-3.1)	3.0 (2.5-3.6)
35.0-39.9	3.6 (3.0- 4.3)	4.4 (3.8-5.1)	3.3 (2.7-3.9)	3.5 (2.9-4.1)	3.9 (3.1-4.8)
≥40.0	4.7 (3.9- 5.6)	5.1 (4.3-6.0)	5.2 (4.3-6.4)	3.5 (2.7-4.5)	2.9 (2.0-4.2)
**Adjusted^b^ population-attributable fraction (95% CI)**					
25.0-29.9	14.5 (11.2-17.7)	15.5 (12.9-18.0)	14.2 (11.1-17.3)	12.2 (9.6-14.9)	20.2 (15.9-24.4)
30.0-34.9	13.7 (11.1-16.2)	15.3 (13.2-17.4)	12.1 (9.9-14.3)	11.2 (9.4-13.1)	18.1 (14.9-21.3)
35.0-39.9	9.3 (7.5-11.1)	10.2 (8.7-11.8)	5.7 (4.3-7.1)	4.7 (3.6-5.8)	8.0 (6.1-10.0)
≥40.0	8.3 (6.7-10.0)	7.1 (5.8-8.4)	5.3 (4.0-6.5)	2.1 (1.4-2.8)	1.9 (0.9-2.9)
Total (≥25.0)	45.8 (40.6-50.9)	48.1 (44.2-52.0)	37.3 (33.0-41.6)	30.3 (26.7-33.9)	48.1 (42.0-54.3)

Abbreviations: BMI, body mass index; CI, confidence interval.

a BMI (kg/m^2^) categories defined by the National Heart, Lung, and Blood Institute (15) as follows: underweight, <18.5; normal weight, 18.5-24.9; overweight, 25.0-29.9; class I obese, 30.0-34.9; class II obese, 35.0-39.9; and class III obese, ≥40.0.

b Models are adjusted for age and parity.
